# Generation of an avian influenza DIVA vaccine with a H3-peptide replacement located at HA2 against both highly and low pathogenic H7N9 virus

**DOI:** 10.1080/21505594.2022.2040190

**Published:** 2022-03-14

**Authors:** Gang Li, Juan Feng, Keji Quan, Zhihao Sun, Yuncong Yin, Yinyan Yin, Sujuan Chen, Tao Qin, Daxin Peng, Xiufan Liu

**Affiliations:** a College of Veterinary Medicine, Yangzhou University, Yangzhou, China; b Jiangsu Co-Innovation Center for the Prevention and Control of Important Animal Infectious Disease and Zoonoses, Yangzhou, China; c The International Joint Laboratory for Cooperation in Agriculture and Agricultural Product Safety, Ministry of Education, Yangzhou University, Yangzhou, China; d College of Medicine, Yangzhou University, Yangzhou, China; e Jiangsu Research Centre of Engineering and Technology for Prevention and Control of Poultry Disease, Yangzhou, China

**Keywords:** Avian influenza virus, H7N9, DIVA, recombinant vaccine, competitive inhibition ELISA

## Abstract

A differentiating infected from vaccinated animals (DIVA) vaccine is an ideal strategy for viral eradication in poultry. Here, according to the emerging highly pathogenic H7N9 avian influenza virus (AIV), a DIVA vaccine strain, named rGD4_HALo-mH3_-TX, was successfully developed, based on a substituted 12 peptide of H3 virus located at HA2. In order to meet with the safety requirement of vaccine production, the multi-basic amino acid located at the HA cleavage site was modified. Meanwhile, six inner viral genes from a H9N2 AIV TX strainwere introduced for increasing viral production. The rGD4_HALo-mH3_-TX strain displayed a similar reproductive ability with rGD4 and low pathogenicity in chickens, suggesting a good productivity and safety. In immuned chickens, rGD4_HALo-mH3_-TX induced a similar antibody level with rGD4 and provided 100% clinical protection and 90% shedding protection against highly pathogenic virus challenge. rGD4_HALo-mH3_-TX strain also produced a good cross-protection against low pathogenic AIV JD/17. Moreover, serological DIVA characteristics were evaluated by a successfully established competitive inhibition ELISA based on a 3G10 monoclonal antibody, and the result showed a strong reactivity with antisera of chickens vaccinated with H7 subtype strains but not rGD4_HALo-mH3_-TX. Collectedly, rGD4_HALo-mH3_-TX is a promising DIVA vaccine candidate against both high and low pathogenic H7N9 subtype AIV.

## Introduction

At the beginning of 2013, H7N9 subtype avian influenza virus (AIV), which caused human infection along with five waves in China [1,2], was initially identified as a low pathogenic AIV (LPAIV) to poultry and spread widely in different provinces, especially in live poultry markets [3,4]. Since the second half of 2016, H7N9 subtype AIV evolved into highly pathogenic AIVs (HPAIV), confirmed by a multi-basic amino acid motif appearing at the cleavage site of HA [5]. Subsequently, H7N9 subtype HPAIV caused outbreaks in chickens, leading to a large number of chicken deaths in several provinces in China [2]. At present, vaccination has become one of the effective strategies to control avian influenza. After the implementation of the national vaccination program with the H5/H7 bivalent and trivalent inactivated vaccines, the prevalence of H7N9 virus in poultry was dramatically limited [6], more importantly, the vaccination of poultry successfully prevented the emergence of new waves of human H7N9 infection [7].

Avian influenza is an important target for eradication, which was listed at the national medium and long-term plan for prevention and control of animal epidemic devised by the government of China [8]. Pathogenic surveillance is an ideal screening strategy, however, diagnostic sensitivity is a high requirement and the monitoring window period is very short, which limit the screening of positive poultry. Serological studies can detect the infection with influenza virus in the absence of symptoms or positive viral characterizations [^9–11^]. In the influenza serological assays, such as hemagglutination inhibition (HI) or microneutralization (MN), the quantification of virus-specific antibodies can be as an indicator of infection [12]. However, serological monitoring can be affected after vaccination. Therefore, it is necessary to design a novel vaccine that distinguishes infected from vaccinated animals (DIVA). This strategy has been successfully applied to the prevention and control of pseudorabies. Since 1990, pseudorabies has been eradicated in some farms and regions by using DIVA strategy based on gE-deleted vaccines and the matched diagnostic kits, such as gE-ELISA kits, which can effectively detect specific antibodies induced by wide type virus [^13–15^]. Several DIVA strategies have been designed for avian influenza vaccines. Nonstructural protein 1 (NS1) that is only detected in infected cells was considered as a target in the design of DIVA vaccines; however, NS1 antibodies is too low to hardly detection [16]. Neuraminidase (NA) is another design target based on different NA subtypes between vaccine strain and epidemic strain [17], whereas possible failures cannot be ignored because continual emerging viruses has a higher probability to acquire similar NA subtype with vaccine strains.

Hemagglutinin (HA) protein is a glycoprotein located on the AIV envelope membrane, which can induce protective neutralizing antibodies. The current vaccines mainly induce neutralizing antibodies against the HA1 protein, which can provide a very effective protection against homologous strains. However, the epitopes located at HA1 protein are prone to mutate, and the vaccine needs to be updated continuously to guarantee the immune efficacy. HA2 protein, located at the stem of HA, has high homology among different subtypes of AIV and chimeric recombination is not prone to occur [18]. If the conservative peptides can be screened at the HA2 protein and replaced with the corresponding peptides of other subtypes of viruses, the DIVA strategy will be possible. Therefore, HA2, as a candidate target for DIVA vaccine design, is very promising.

Previously, we successfully screened a specific epitope on the HA2 protein of H7N9 subtype AIV, named H7–12 peptide, based on peptide microarray technology. Subsequently, a serological DIVA vaccine was developed by using the chimeric HA epitope approach based on LPAIV JD/17 strain [19]. With the highly pathogenic H7N9 strain became the dominant epidemic strain, therefore, a H7N9 avian influenza DIVA vaccine was successfully developed based on substituted 12 peptides of H3 subtype influenza virus located at HA2 in this study. In addition, the multi-basic amino acid motif located at the HA cleavage site was removed for attenuation to ensure the safety of vaccine production, and a virus skeleton from a H9N2 subtype AIV (TX strain) was introduced for increasing viral production. Furthermore, vaccine immune protection against H7N9 subtype LPAIV and HPAIV were also evaluated. The DIVA properties can be easily detected by our established competitive inhibition ELISA method based on 3G10 McAb, which is a more suitable method for wide application in production practice.

## Materials and methods

### Biosafety and animal care

3- and 6-week-old specific pathogen-free (SPF) chickens were purchased from Sinopharm Yangzhou Weike Biological Engineering Co., Ltd. All experiments involving H7 viruses were approved by the Institutional Biosafety Committee of Yangzhou University and were performed in animal biosafety level 3 (ABSL-3) facilities according to the institutional biosafety manual (CNAS BL0015). The protocols of all animal studies were approved by Jiangsu Province Administrative Committee for Laboratory Animals (approval number: SYXK-SU-2016–0020) and complied with the guidelines of Jiangsu Province Laboratory Animal Welfare and the ethics of Jiangsu Province Administrative Committee of Laboratory Animals.

### Viruses, cells, and plasmids

A total of 15 AIV strains were isolated from live poultry markets or diseased chickens by our laboratory (Table S1), including ten different subtypes of AIVs (H1N1, H3N2, H4N6, H5N2, H5N6, H5N1, H6N2, H7N9, H9N2, and H10N3), and antiserum against each virus was prepared from SPF chickens immunized with whole inactivated virus. Viruses were propagated in 10-day-old SPF embryonated chicken eggs (ECEs) at 37°C and the viral allantoic fluids were collected and stored at −80°C. Viral infectivity of each strain was determined by serial titration in 10-day-old ECEs, and was calculated as 50% of the egg infective dose (EID_50_)/.1 mL by the Reed-Muench method [20]. Madin-Darby canine kidney (MDCK) cells and human embryonic kidney (293T) cells were maintained in Dulbecco’s Modified Eagle’s Medium (HyClone, USA) supplemented with 10% fetal bovine serum (Gibco, USA) at 37°C in an atmosphere of 5% CO_2_. Chicken embryo fibroblast (CEF) cells were prepared and maintained in M199 medium (HyClone, USA) with 4% fetal bovine serum. The eight plasmids (HA, NA, PB2, PB1, PA, NP, M, and NS based on pHW2000 vector) of TX strain were previously constructed [21] and kept in our laboratory.

### Generation of antiserum

The viral allantoic fluids were inactivated by mixing .1% formaldehyde solution at 4℃ for 24 h, and then the inactivation level was tested by performing serial passages on eggs. The completed inactivated virus was emulsified by white oil adjuvant (Sinopharm-vacbio, Yangzhou, China) for preparing inactivated vaccines. Immune antiserum was generated by vaccinating 21-day-old SPF chickens (n = 5) with the inactivated vaccine and identified by hemagglutination inhibition (HI) assays. For preparation of infected antiserum, 7-week-old SPF chickens (n = 3) were infected intranasally with GD4 strain with 10^4^EID_50_/.1 mL. After 14 d. p. i., infected antiserum was generated after HA titer ≥7. 3-week-old SPF chickens (n = 5) were infected intranasally with JD/17 strain with 10^6^EID_50_/.1 mL. After 21 d. p. i., infected antiserum was generated after HA titer ≥7.

### Phylogenetic analyses

HA gene sequences from JD/17, XZ-1, GD4, HZLH2, and XT-3 were obtained by sequencing, and the gene sequences from all other viruses were obtained from GISAID and GenBank. The complete sequences were further chosen to perform multiple sequence alignment by MEGA (Version 6), and then maximum likelihood phylogenetic trees were inferred with 1000 bootstraps.

### Thermostability and pH stability

For thermostability assay, all viruses were diluted to the same EID_50_ and then incubated in water bath at 37℃ or 42℃. At day 1, 3, and 5. The HA titer of virus was determined. For 56℃ thermostability, all viruses were incubated in water bath at 56℃ for 0, 5, 10, 15, 30, 60, or 90 min, and then the treated samples were quickly placed at 4℃ for 5 min, and the HA titer was determined. The pH stability was assayed as previously described [22]. In brief, viruses were mixed with an equal volume buffer with different pH levels and incubated at 37℃ for 10 min. The titers of all samples were determined using hemagglutination assay with 1% chicken red blood cells.

### Construction of a H7N9 rGD4_HAlo-mH3-_TX strain

Briefly, the HA gene of GD4 with removed the multi-basic amino acid motif was amplified by overlapping PCR using primer pairs, including HA_Lo_-1-F, HA_Lo_-1-R, HA_Lo_-2-F, and HA_Lo_-2-R. Furthermore, HA_Lo_ gene of GD4 virus with substitution of H3 subtype 12 peptide at HA2 was generated by overlapping PCR using primer pairs, including HA_Lo-mH3_-1-F, HA_Lo-mH3_-1-R, HA_Lo-mH3_-2-F, HA_Lo-mH3_-2-R, HA_Lo-mH3_-3-F, and HA_Lo-mH3_-3-R (Table 1). Next, the HA_Lo-mH3_ was cloned into pHW2000 vector, which was combined with the NA plasmids from GD4 and high-yield inner viral backbone from H9N2 subtype TX strain (containing PB2, PB1, PA, NP, M, and NS plasmid) to generate a recombinant strain, named rGD4_HALo-mH3_-TX, by a reverse genetics method as described previously [23]. Sequencing results showed that all the viral genes were genetically stable without any unwanted mutation.

**Table 1.** Primers for construction of the recombination HA gene

Gene segment	Primer names	Primer sequence (5'-3')
HA_Lo_-1	1-F	TATT*CGTCTC*AGGGAGCRAAAGCAGGGG
	1-R	CAAATAGGCCTCTTCCCTTTGGAACCTCAGGAA
HA_Lo_-2	2-F	AGGTTCCAAAGGGAAGAGGCCTATTTGGTGCTAT
	2-R	ATAT*CGTCTC*GTATTAGTAGAAACAAGGGTGTTTT
HA_Lo-mH3_-1	1-F	TATT*CGTCTC*AGGGAGCRAAAGCAGGGG
	1-R	TGTTTTTTCAAACAGTTTGTTCATTTCTGAGTCAGC
HA_Lo-mH3_-2	2-F	TATT*CGTCTC*AGGGAGCRAAAGCAGGGG
	2-R	CCCTCAGTTGCTTCTTTGTTTTTTCAAACAGTTT
HA_Lo-mH3_-3	3-F	AAGAAGCAACTGAGGGAAAATGCTGAAGAAGATGG
	3-R	ATAT*CGTCTC*GTATTAGTAGAAACAAGGGTGTTTT

The italic represents the restriction endonuclease *BsmbI* site; Underline represents the changed sequence.

### IVPI assay

Fresh infective allantoic fluid was diluted 1:10 in sterile phosphate buffer saline (PBS). A sample (.1 mL) of the diluted virus was injected intravenously into each of 6-week-old SPF chickens (n = 10). All of the chickens were examined daily for 10 days and scored based on the condition of each chicken as described in the OIE Manual [24].

### Vaccination and challenge assay

3-week-old SPF chickens (n = 10) were injected via neck subcutaneous with .3 mL each formalin-inactivated vaccine (rGD4_HALo-mH3_-TX or rGD4, 10^6^EID_50_/.1 mL) or .3 mL of PBS as controls. Every week after vaccination, serum from all chickens was collected for testing. Three weeks after vaccination, the chickens were challenged intranasally with 10^6^ EID_50_ of H7 virus (GD4 or JD/17). Oropharyngeal and cloacal swabs were collected at 1, 3, and 5 days post-challenge for the detection of virus shedding. All chickens were observed for signs of disease or death during 14 days after challenge.

### Preparation of McAb

Four 6-week-old SPF BALB/c mice were subcutaneously injected with 50 μg H7–12 peptide-BSA conjugates that were emulsified with the same volume of Freund’s complete adjuvant (Sigma Aldrich, St. Louis, MO, USA). After three weeks, the mice were immunized by subcutaneous injection of H7–12 peptide-BSA with the same volume of Freund’s incomplete adjuvant (Sigma Aldrich, St. Louis, MO, USA). The HI titer of serum isolated from the immunized mice was determined. Once a high titer was obtained, the spleen cells were isolated and fused with the cultured SP2/0 myeloma cells at ratio of 5:1 in the presence of 1 mL PEG1500 (Roche, Switzerland) for preparing hybridoma cells [25]; The supernatant of cell culture from individual wells containing hybridoma cells was collected and detected after 10 d by indirect ELISA (iELISA) as described [26]. After limiting dilution and three subclonal screenings, the hybridoma cells with high affinity and specificity against H7–12 peptide were injected intraperitoneally into the BALB/c mice for the production of McAb in ascites [27], and then the soluble IgG antibodies were purified.

### IFA

CEF cells were infected with different AIVs at multiplicity of infection (MOI) = 10, and cultured for 12 h at 37℃. Then, the cells were fixed with cold methanol, after washing with PBS, the plates were incubated with monoclonal antibody (3G10, 1:2000) against H7–12 peptide for 1.5 h at 37℃, then rinsed three times for 5 min. The 1:5000 Alexa Fluor^TM^ 488 goat anti-mouse IgG (H+L) (ThermoFisher, USA) were incubated at 37℃ for 1 h. Next, the plates were rinsed three times and visualized with a fluorescence microscopy (Olympus, Tokyo, Japan).

### Competitive inhibition ELISA

ELISA plates were coated with H7–12 peptide (50 µg/mL, GL Biochem Ltd, China) overnight at 4°C Then, the plates were blocked for 2 h at 37℃ with 5% nonfat milk. Infection or immune serum were prediluted 1:4, and incubated on the plates for 1 h at 37℃. After extensive washing three times, the plates were incubated for 1 h at 37℃ with an enzyme-labeled monoclonal antibody (3G10) were prediluted 1:200 (100 μL/well). After three washings, the plates were overlaid with 3,3,'5,5'-tetramethylbenzidine (TMB, Beyotime Biotechnology, China). Reactions were stopped by using 2 M H_2_SO_4_. Optical density (OD) was read at 450 nm of each well. Inhibition rate (I) = (1-OD_450_ positive value(P)/negative value(N)) × 100%. 30 negative serums were detected by competitive inhibition ELISA. The average (×) of the inhibition rate of 30 negative serums was 8.17 and the standard deviation (SD) was 4.03. X + 2 SD was as the negative cutoff value, which was 16.14%; X + 3SD was as the positive cutoff value, which was 20.23%. The value between 16.14% and 20.23% was suspicious and needs to be tested again.

### Statistical analysis

Results were expressed as the means ± standard deviations (SD). A one-way ANOVA analysis of variance was employed to compare the variance between the different groups. *P < .05 , **P < .01.

## Results

### H7N9 subtype HPAIV shows a far genetic relationship in comparison to H7N9 subtype LPAIV

**Figure 1.**
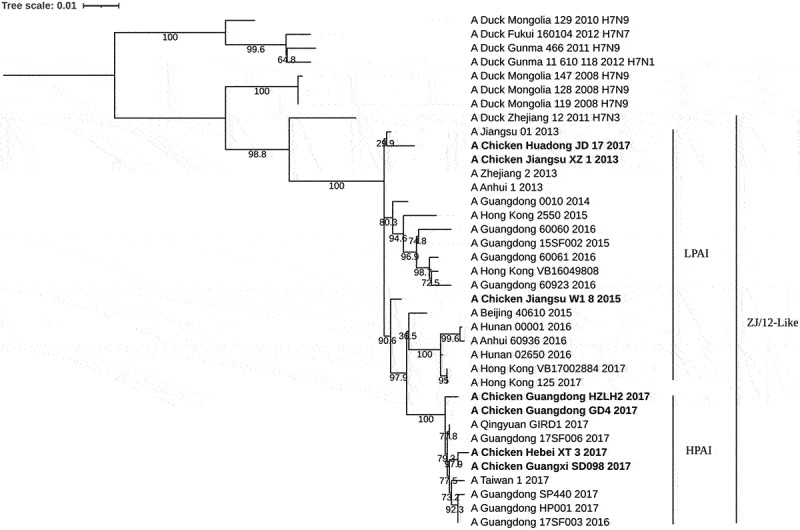
Phylogenetic tree of H7N9 strains based on HA gene. The chosen LPAIV and HPAIV strains were confirmed based on their law of basic amino acid motif located at the HA cleavage site. A/Chicken/Guangxi/SD098/2017 strain is the HA gene donor virus of H7-Re2.

The phylogenetic tree showed that the mean genetic distance of HA in the ingroup of HPAIV and LPAIV was .004 and .018, respectively. The average genetic distance of HA among groups between HPAIV and LPAIV was .026 (Figure 1). These results showed that GD4, HZLH2, XT-3, and SD098, belonged to HPAIV, have a far genetic distance from LPAIV. Therefore, it is necessary to develop a novel vaccine against both HPAIV and LPAIV.

### Rescue of rGD4_HAlo-mH3_-TX virus

**Figure 2.**
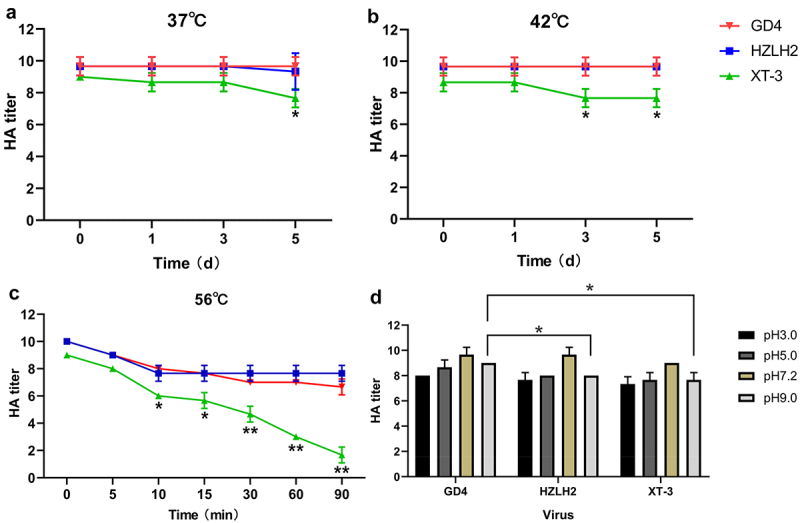
Thermal and pH stability of H7N9 viruses. H7N9 GD4, HZLH2, and XT-3 strains were incubated at 37°C (a) or 42°C (b) for 5 days, or at 56°C (c) for 90 mins. The HA titers were determined. (d) for pH stability, the viruses were incubated in each buffer at 37°C for 10 min, and the HA titers were then determined. The data are presented as the mean ± SD. *P <.05, **P <.01.

**Figure 3.**
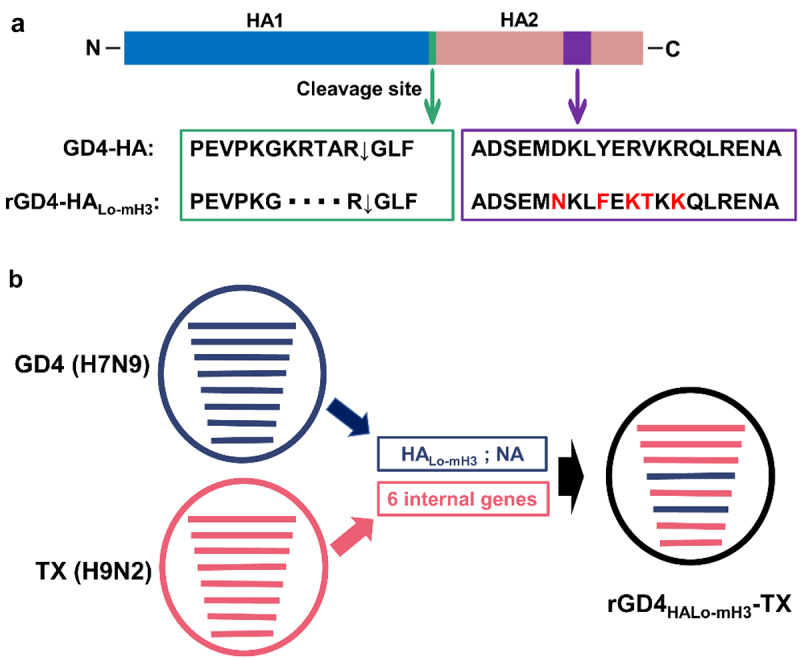
Construction of recombinant virus. (a) the multi-basic amino acid motif located at the HA cleavage site were removed and the HA2 specific peptide (^463^ADSEMDKLYERVKRQLRENA^482^) were replaced by H3 subtype 12 peptide (^463^ADSEMNKLFEKTKKQLRENA^482^). (b) the recombinant HA_Lo-mH3_ and the NA plasmid of GD4, combined with high-yield viral backbone from H9N2 subtype TX strain (containing PB2, PB1, PA, NP, M, and NS plasmids), was used to construct a recombinant virus based on our established reverse genetic manipulation.

The 50% tissue infective doses (TCID_50_) in chicken embryo fibroblast (CEF) cells and 50% egg infectious doses (EID_50_) of 3 HPAIVs, including GD4, HZLH2 (A/Chicken/Guangdong/HZLH2/2017, H7N9), and XT-3 (A/Chicken/Hebei/XT-3/2017, H7N9), were tested for evaluating the basic biological characteristics of the strains. The results showed that GD4 had higher TCID_50_ and EID_50_ titer compared with other two strains (Table 2). Furthermore, the thermostability and pH stability of viruses were performed for evaluate the viral stability. We found that GD4 and HZLH2 showed a higher thermostability at 37℃, 42℃, or 56℃ compared with XT-3 strain (Figure 2(a-c)). Meanwhile, the GD4 of pH stability displayed a highest level in comparison with other strains (Figure 2(d)). Therefore, GD4 was considered to develop a recombinant vaccine candidate strain by reverse genetic technology. The multi-basic amino acid motif located at the HA cleavage site was removed for attenuation and the H7–12 peptide (^463^ADSEMDKLYERVKRQLRENA^482^) located at HA2 of GD4 was replaced by H3 subtype 12 peptide (^463^ADSEMNKLFEKTKKQLRENA^482^), which is as a marker for DIVA strategy (Figure 3(a)). The recombinant HA fragment, named the HA_Lo-mH3_, combined with the NA plasmids from GD4 and high-yield viral backbone from H9N2 subtype TX strain (containing PB2, PB1, PA, NP, M, and NS plasmids), was used to construct a recombinant virus based on our established reverse genetic manipulation [22], named rGD4_HALo-mH3_-TX (Figure 3(b)). Sequencing results showed that all the virus genes were genetically stable without any unwanted mutation after passaging 5.

**Table 2.** The TCID_50_ and EID_50_ of H7N9 strains

Virus	TCID_50_ (log_10_/0.1 mL)	EID_50_ (log_10_/0.1 mL)
GD4	7.33	8.17
HZLH2	6.67	8.00
XT-3	6.00	7.83

### rGD4_HAlo-mH3_-TX strain displays a good reproductive ability and low pathogenicity

After removing the multi-basic amino acid motif located at the HA cleavage site, the EID_50_ titer of rGD4_HALo_ (8.0 log_10_/.1 mL) was lower than that of rGD4 (8.5 log_10_/.1 mL). Notably, after substitution of a high-yield viral backbone from H9N2 subtype TX strain, The EID_50_ titer of rGD4_HALo-mH3_-TX strain were same with that of rGD4 (8.5 log_10_/.1 mL). Meanwhile, The HA titer of rGD4_HALo-mH3_-TX maintained a high level (10 log_2_). These data suggested that our vaccine design strategy still maintain a good viral reproductive ability (Table 3). In the chicken embryo infection assay, rGD4 induced mortalities of embryos within 36 h, while the chicken embryos infected with rGD4_HALo_ or rGD4_HALo-mH3_-TX were still alive at least 120 h. Furthermore, chicken intravenous pathogenicity index (IVPI) assay was introduced to evaluate the pathogenicity *in vivo*. Chickens inoculated with rGD4, rGD4_HALo_ or rGD4_HALo-mH3_-TX via the intravenous route died 48 h or alive, with 2.55, .10 or .01 IVPI values, respectively. The above results indicated that the pathogenicity of the rGD4_HALo-mH3_-TX has been weakened significantly.

**Table 3.** Viral reproductive ability and pathogenicity

Viruses	HA titer (log_2_)	EID_50_(log_10_/0.1mL)	MDT^a^	IVPI^b^
rGD4	11	8.50	36 h	2.55
rGD4_HALo_	10	8.00	>120 h	0.10
rGD4_HALo-mH3_-TX	10	8.50	>120 h	0.01

^a^Mean death time (MDT), 10-day-old SPF ECEs were used for the assay of infection.

^b^6-week-old SPF chickens were used for the assay of IVPI.

### rGD4_HAlo-mH3_-TX strain shows a good immune efficacy and cross-immunoprotection against H7N9 subtype HPAIV and LPAIV

**Figure 4.**
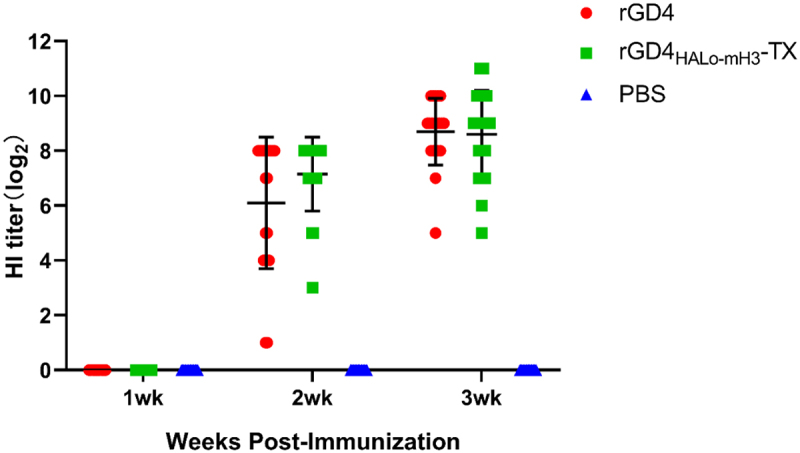
Serum HI level of each group after first immunization.

Three-week-old SPF chickens were immunized with inactivated rGD4 or rGD4_HALo-mH3_-TX virus and the protective efficacy was evaluated. The HI titers (log_2_) of rGD4 and rGD4_HALo-mH3_-TX immune serum against GD4 were 6.10 ± 1.57 and 7.15 ± 1.63, respectively, at 2 weeks after first immunization, and the titers rose to 8.70 ± 1.22 and 8.60 ± 1.60, respectively, at 3 weeks post first immunization (Figure 4). The result of cross-antibody titers showed that the average HI titers (log_2_) of vaccinated chickens with rGD4 or rGD4_HALo-mH3_-TX against JD/17 (6.85 ± 1.63 or 7.25 ± 1.55, respectively) were low than that against GD4 (8.70 ± 1.22 or 8.60 ± 1.60, respectively) at 3 weeks after first immunization (Table 4). These results indicated that rGD4_HALo-mH3_-TX can induce similar antibody levels with rGD4 and also can produce good cross-antibody levels against LPAIV JD/17. Next, chickens were challenged with 10^6^ EID_50_ of HPAIV (GD4) strain at 3-week post-vaccination, and all PBS-vaccinated chickens died within 5 days. All vaccinated chickens with rGD4_HALo-mH3_-TX and rGD4 showed a 100% clinical protection. Of note, two chickens vaccinated with rGD4 appeared cloaca shedding and one chicken had oropharyngeal shedding (80% shedding protection), whereas no cloaca shedding and low oropharyngeal shedding (1/10) in the chickens vaccinated with rGD4_HALo-mH3_-TX (90% shedding protection) were detected. After challenge with 10^6^ EID_50_ of heterologous H7 LPAIV (JD/17), all PBS-vaccinated chickens shed virus within 5 days. All vaccinated chickens with rGD4_HALo-mH3_-TX or rGD4 showed no clinical signs during observation. The chickens vaccinated with rGD4_HALo-mH3_-TX had a higher shedding protection (80%) than rGD4 immunization group (70%). These data suggested that rGD4_HALo-mH3_-TX showed a good immune efficacy and cross-immunoprotection against H7N9 subtype HPAIV and LPAIV.

**Table 4.** Survival and shedding of immunized chickens after challenge

Immunized groups	HI titer(log_2_)	Challenge virus	Positive samples/Total samples						Shedding protection(%)	Mortality(%)
			1 d.p.c.		3 d.p.c.		5 d.p.c.			
			O^c^	C^d^	O	C	O	C		
rGD4	8.70^a^(±1.22)	GD4	0/10	0/10	0/10	0/10	1/10	2/10	80	0
	6.85^b^(±1.63)	JD/17	0/10	0/10	2/10	0/10	3/10	2/10	70	0
rGD4_HALo-_ _mH3-NA_-TX	8.60^a^(±1.60)	GD4	0/10	0/10	0/10	0/10	1/10	0/10	90	0
	7.25^b^(±1.55)	JD/17	0/10	0/10	1/10	0/10	2/10	0/10	80	0
PBS	ND^e^	GD4	3/10	0/10	6/6	6/6	NS^f^	NS	0	100
	ND	JD/17	6/10	0/10	8/10	8/10	10/10	6/10	0	0

d.p.c. days post-challenge; ^a^ HI titer against GD4; ^b^ HI titer against JD/17; ^c^ Oropharyngeal swab; ^d^ Cloacal swab; ^e^ No detection; ^f^ No survivors.

## 3G10 monoclonal antibody shows a good specificity for identification of H7N9 subtype HPAIV and LPAIV

**Figure 5.**
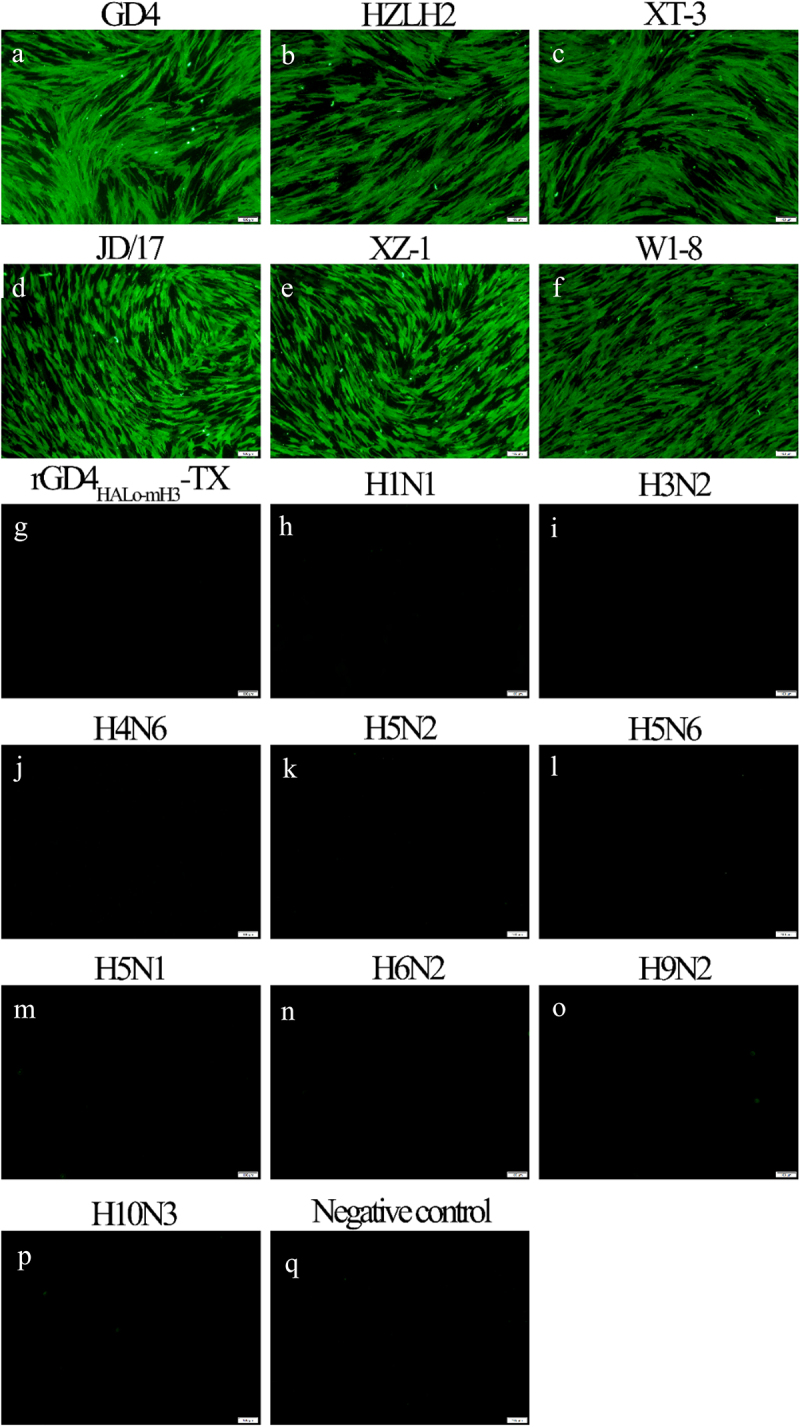
Specificity of McAb 3G10 by IFA. (a-p) CEF cells were infected with different AIVs, including GD4, HZLH2, XT-3, JD/17; XZ-1, W1–8, rGd4_halo-mH3_-TX, H1N1, H3N2, H4N6, H5N2, H5N6, H5N1, H6N2, H9N2, and H10N3 subtype AIVs. q: Negative control. IFA was performed based on 3G10 McAb. Scale bar = 100 μm.

The monoclonal antibody (McAb), named 3G10, against H7–12 peptide was successfully prepared. The 3G10 was used as the primary antibody to perform the immunofluorescence assay (IFA). CEF cells infected with both H7N9 subtype HPAIVs (GD4, HZLH2, and XT-3) and LPAIVs (JD/17, XZ-1, and W1–8) were positively responded to 3G10 (Figure 5(a-f)), while rGD4_HALo-mH3_-TX and other 9 subtype AIVs (Table S1) were negatively responded with 3G10 (Figure 5(h-p)), indicating that 3G10 showed a good specificity to identify H7N9 subtype HPAIV and LPAIV. Importantly, rGD4_HALo-mH3_-TX lost its specific binding capacity with 3G10 after the H3–12 peptide was replaced, implying that 3G10 possessed the ability to distinguish the H7N9 subtype wild-type viruses from a rGD4_HALo-mH3_-TX candidate strain.

## rGD4_HAlo-mH3_-TX strain shows a good DIVA property by using a matched competitive inhibition ELISA

The established competitive inhibition ELISA method based on 3G10 McAb was used to detect H1, H3, H4, H5 (Re-8, Re-11, and Re-12), H6, H7 (H7-Re2), H9, and H10 positive serums and negative serum. Meanwhile, GD4, JD/17, and rGD4_HALo-mH3_-TX immune or infected serums were also detected. As shown in Table 5, the inhibition rate of H7-Re2, GD4, and JD/17 immune serum was 48.56 ± 3.98%, 52.24 ± 1.83%, and 54.87 ± 2.81%, respectively, which is higher than the negative value of 16.14%, while the serum inhibition rate of other HA subtypes is less than 16.14%, demonstrating that the established competitive inhibition ELISA method showed a good specificity to identify H7 subtype wild type virus. In addition, the inhibition rate of GD4 and JD/17 infected serum was 56.79 ± 3.76 and 55.33 ± 3.06, respectively, which showed a strongly positive response and indicated that the simulated infection serums can also be accurately detected by this method. Importantly, the inhibition rate of rGD4 HA_Lo-mH3_-TX immune serum was 4.89 ± 1.43%, implying an ideal DIVA property for distinguishing between vaccine strains and wild-type strains.

**Table 5.** The specificity of established competitive inhibition ELISA

Serum	Inhibition rate (%, mean±SD)	Serum	Inhibition rate (%, mean±SD)
H1	7.02 ± 1.29	H7-Re2	48.56 ± 3.98
H3	9.72 ± 3.15	H7-GD4 (Immune)	52.24 ± 1.83
H4	6.58 ± 3.71	H7-JD/17 (Immune)	54.87 ± 2.81
H5 (Re-8)	4.57 ± 2.87	H7-GD4 (Infected)	56.79 ± 3.76
H5 (Re-11)	11.45 ± 3.59	H7-JD/17 (Infected)	55.33 ± 3.06
H5 (Re-12)	6.55 ± 1.03	H7-rGD4_HALo-mH3_-TX	4.89 ± 1.43
H6	10.61 ± .81		
H9	9.62 ± 1.18		
H10	8.28 ± 1.59		

## Discussion

The option of vaccination against AIV is major prevention and control measure in poultry industry. However, traditional vaccines, such as the inactivated whole-virus vaccines, resulted in a malfunction of serologic diagnostic assay for surveillance and identify of infected animals, which is critical way for eradication of epidemic disease. In this study, a novel DIVA vaccine was developed with a good immune efficacy and cross-immunoprotection against H7N9 subtype HPAIV and LPAIV. In addition, the developed competitive inhibition ELISA method based on 3G10 McAb with a good specificity can accurately distinguish between immune serum of rGD4_HALo-mH3_-TX and infected serum of wild type H7N9 strains.

The current influenza vaccines mainly induce anti-HA antibodies, which specifically target the antigenic sites located at the globular head domain of the HA1 region for blocking receptor binding [28,29]. However, viral mutations occur frequently at HA1 region, which resulted in a low neutralizing activity of antibodies induced by influenza vaccines. Compared with HA1, HA2 is more conservative and gradually become a main target protein in the study of universal epitopes [30], which implying that a potentially conserved peptide could be chosen as a DIVA marker. The results of IFA experiments also showed the 3G10 McAb against our H7–12 peptide located at HA2 only responded with cells infected by H7 subtype AIVs but not that infected by other 9 subtype AIVs, including H1, H3, H4, H5 (Re-8, Re-11, and Re-12), H6, H9, and H10 subtype, suggesting H7–12 peptide showed a good specificity for H7 subtype AIVs. Furthermore, 3G10 McAb can well identify both H7 subtype HPAIV and LPAIV despite a far genetic relationship was appeared in recent, indicating that the H7–12 peptide reflected a highly conservative feature in H7 subtype AIVs. In order to achieve the DIVA strategy, H7–12 peptide was replaced by corresponding H3 sequence based on five amino acids difference. A recombinant chimeric influenza virus GD4_HALo-mH3_-TX strain was successfully rescued by reverse genetic technology, implying that only the mutations with five amino acids did not influence viral biological properties. Moreover, 3G10 McAb cannot identify the chimeric virus after replacing H7–12 peptide with five amino acids mutations in IFA assay. These results revealed that rGD4_HALo-mH3_-TX strain is expected to be a marker vaccine strain candidate to distinguish infected from vaccinated animals and 3G10 McAb with good specific and broad-spectrum properties has a promise to be applied in DIVA diagnosis.

The classic virus serological test, such as hemagglutination inhibition or microneutralization, can detect virus-specific antibodies as indicators for monitoring infection [12], however, these methods cannot distinguish between the antibodies induced by vaccination and wild virus infection in the host. Although a peptide microarray for DIVA identification was successfully generated in our previous study, and showed much more sensitive than that by HI test [19], a special equipment is needed currently. In comparison with these serologic methods, ELISA technique is widely regarded as an essential approach superior in accuracy, rapid, and throughput for early diagnosis, shows obvious advantages in wide promotion and apply [31]. Among different kind of ELISA assays, competitive inhibition ELISA, also called epitope-blocking ELISA, can achieve a distinction between immune serum and infected serum. Therefore, a highly specific McAb is necessary to recognize a broadly conserved antigenic epitope throughout H7 subtype strains which can consistently induce antibody response in infected but not rGD4_HALo-mH3_-TX vaccinated hosts. Our generated 3G10 McAb-based competitive inhibition ELISA method showed that only the serum against H7 subtype HPAIV or LPAIV wild type has a positive inhibition rate, whereas the serum against the chimeric virus rGD4_HALo-mH3_-TX has a typical negative inhibition rate, demonstrating that 3G10 McAb showed a high specificity and broad-spectrum for covering both H7 subtype HPAIV and LPAIV, and the 3G10 McAb-based competitive inhibition ELISA showed an excellent capacity for DIVA diagnosis.

H7N9 subtype AIV is an important zoonotic pathogen, thus the safety of vaccine production also needs to be considered. HPAIVs usually contains continuous basic amino acids at the cleavage site of the HA gene. Our previous study successfully achieved the attenuation of H5N1 subtype HPAIV based on modifying basic amino acids at the cleavage site of the HA gene [32]. Meanwhile, the attenuation strategy might be resulted in the decline of virus production, thus, replacement of the internal backbone with high-yield property is also an important solution. Subbarao et al. [33]. removed the multibasic amino acid motif in the HA gene of epidemic strains and transfected a recombinant virus from in a background of internal genes derived from PR8 for the first time, and a new attenuated recombinant candidate strain was successfully developed. H7N9 viruses initially reported in 2013 were generated on the basis of complete internal genes from H9N2 subtype AIVs [34,35], implying that the internal backbone of H9N2 virus is better matching the H7N9 virus. We previous found that TX is a natural attenuated strain of the avian H9N2 subtype and possesses a strong virus replication capacity in chick embryo [36], implying its internal backbone could be used for virus production. Therefore, based on the consideration of production safety and high yield, the multi-basic amino acid motif located at the HA cleavage site was removed and the TX internal backbone was replaced successfully into H7N9 GD4 strain. Our constructed rGD4_HALo-mH3_-TX recombinant strain still maintained a comparable viral reproductive ability with rGD4 and showed a low pathogenicity that chicken intravenous pathogenicity index (IVPI) remarkably decreased to .01.

Immune efficacy is unaffected in the design of DIVA vaccine, which is also needed to considered. Our designed the rGD4_HALo-mH3_-TX strain shows a minimal mutation with five amino acids at HA2, but not HA1, thus the strain can induce a similar antibody level with rGD4, indicating that the substitution of at HA2 did not affect the ability of antibody production, which also overcomes the disadvantage of poor antibody inducibility of DIVA vaccine [37,38]. In addition, rGD4_HALo-mH3_-TX strain is well matched currently epidemiological characteristics of highly pathogenic H7N9 strains in China, and provided 100% clinical protection and 90% shedding protection against HPAIV GD4. Meanwhile, rGD4_HALo-mH3_-TX strain also produced good cross-antibody level and 80% shedding protection against LPAIV JD/17, which is comparable with rGD4 strain. The data suggested that HA2 replacement did not affected the cross-protection capacity of rGD4_HALo-mH3_-TX strain against both high and low pathogenic H7N9 virus. Despite the avian influenza viruses shows a rapid evolution, our research platform based on a novel DIVA vaccine strategy can realize rapid vaccine updating by HA modification for matching epidemic strains.

Taken together, we successfully developed a rGD4_HALo-mH3_-TX-based inactivated H7N9 recombinant vaccine with high safety, high-yield, and high immune efficacy. Moreover, DIVA strategy was also successfully designed based on the replacement of H7–12 peptide at HA2, and the infection serum of wild type strains and the vaccine immune serum can be accurately distinguished by our established 3G10 McAb-based competitive inhibition ELISA method, which will be expected to push the eradication process of H7N9 subtype avian influenza.

## Data Availability

All data are available from the authors upon reasonable request.
